# Notes and News

**Published:** 1896-08-22

**Authors:** 


### NOTES AND NEWS.

(COLLECTED AND COMMUNICATED.)

GLOUCESTER is to have a new infectious diseases hospital. A site has been purchased for £3,500.

THE Liverpool Medical Institution will give a banquet in honour of Sir Joseph Lister on September 19th.

THE Royal Victoria Hospital, Montreal, has been permanently endowed by Lord Mount-Stephen and Sir Donald Smith with 40,000 dollars.

SIR DINSHAW MANECKJEE PETIT intends building a hospital for Parsees in Bombay. He has already bought a site on the Khumballa Hill.

THE Home Secretary has received a petition signed by 8,000 persons, from the Chelsea and Pimlico Protest Committee, against the licensing of the British Institute of Preventive Medicine for vivisection purposes.

THE report of the Medical Officer of Health for Gloucester, issued on August 7th, states that no fresh case of small-pox has occurred for fourteen days previously, and the general state of health appears to be good.

A NEW isolation hospital has been opened at Blackbridge, near Malmesbury. The hospital is a wooden structure, which can contain twelve to twenty beds, and near it is a cottage to be used by the nurses when required. The scheme has been carried out principally through the energy of Mr. John Alexander, a County Council Alderman and Guardian of the Poor.

WE learn that a sum of £1,250 has been received from the subscribers to the erection of the Working Men's Institute, at Moffat, for the endowment of a cottage hospital in the burgh. When the institute was sold to the Proudfoot trustees, the Court of Session determined that the money was divisible amongst the subscribers, who were asked by the trustees of the Working Men's Institute to devote the whole amount to the proposed cottage hospital. That request was almost unanimously complied with, so that after deduction of legal and other expenses the above sum has been received.

IT is proposed to entirely remodel the Presidency General Hospital at Calcutta. The Public Works Department have reported unfavourably of the hospital as it now stands; and, as it is believed that funds will be forthcoming without much difficulty, a committee has the subject under consideration.

THE recent action on the part of the Stepney Guardians is a distinct advance in the cause of humanity. They have adopted a plan for providing medical assistance for the poor in emergency by consenting to pay the fee of a duly qualified medical man who might be called to attend in such a case.

THE Metropolitan Convalescent Institution has received, through Mr. George Herring, a donation of £400 from the Baroness de Hirsch. The North-West Hospital has received £1,000, the St. Mary's Hospital, Paddington, £500, St. George's Hospital £500, and the London Lock Hospital and Rescue Home, Harrow-road, £200, from the same source.

IT is not generally known that dogs can be utilised in times of war. It appears, however, that the Germans have found that they can be very useful in the ambulance service. They can be trained to seek the wounded, whom they find with unfailing certainty, and conduct the ambulance men to the injured whom they have discovered.

A NEW infectious diseases hospital has been opened at Newport. It stands on a site of five acres, in an isolated situation. The building consists of administrative block, two fever pavilions, isolation block, laundry, porter's lodge, and mortuary. Each pavilion contains two wards for six beds in each. The total cost has been £14,000. The buildings are of brick, with Bath stone dressings.

LORD WOLSELEY paid his first official visit to Netley Hospital at the end of last month, and then unveiled a portrait of Surgeon-General Sir Joseph Fayrer. Later Lord Wolseley presented prizes to the students of the Army Medical School, and made an appropriate speech. The Director-General of the Army Medical Department, Professor Lane Notter, read a very favourable report from the Examiners.

THE fifteenth Congress of the Sanitary Institute will be held at Newcastle, commencing September 2nd, under the presidency of the Right Hon. the Earl Percy. A health exhibition to be held in connection with the Congress, will be opened by the Duke of Cambridge on September 2nd. Over 160 authorities, including several county councils, have appointed delegates to the Congress. Very excellent and cheap railway arrangements have been made by the Midland Railway Company.

IF there are those still remaining who require to be convinced that sanitary measures have definite and beneficial results they should study the address of the Lord Provost of Glasgow at the recent annual congress of the British Institute of Public Health, held in that City. Carefully-prepared statistics illustrate the Lord Provost's contentions, and he quoted Dr. Chalmer's life table, which shows that a citizen of Glasgow to-day of ten years of age has a better expectation of life by six years than one of that age living fifty years ago.

